# More than intentions: Importance of motherhood predicts first but not subsequent births

**DOI:** 10.1016/j.alcr.2025.100708

**Published:** 2025-11-09

**Authors:** Arthur L. Greil, Karina M. Shreffler, Stacy M. Tiemeyer, Julia McQuillan

**Affiliations:** a Division of Social Sciences, Alfred University, United States; b Fran and Earl Ziegler College of Nursing, University of Oklahoma Health Sciences Center, United States; c Department of Sociology, University of Nebraska, Lincoln, United States

**Keywords:** Birth, Fertility, Fertility intentions, Importance of motherhood, Parity

## Abstract

Even though there is a strong association between fertility intentions and fertility outcomes, fertility intentions do not consistently predict fertility outcomes, leading to questions about the utility of the concept. Our goal is to assess whether a measure of importance of motherhood could replace or add to fertility intentions, and whether there is a difference between predicting first or subsequent births. Using the two-wave National Survey of Fertility Barriers (women initially ages 25–45, *n* = 1381), this study applies sequential logistic regression analysis with missing data imputation to explore associations of prospective fertility intentions and importance of motherhood with subsequent births among women. Results indicate that both fertility intentions and importance of motherhood have independent associations with subsequent births among nulliparous women, but importance of motherhood does not predict having a birth among women who already have at least one child. This suggests that importance of motherhood contributes beyond fertility intentions to understanding whether nulliparous women have a child or remain childless, but not to understanding achieved fertility.

## 1. Introduction

Accurate predictions of fertility intentions and pregnancies are important for population projections, maternal and infant health support, and estimates for national resources (Shreffler, 2024). There has been considerable research on fertility outcomes using theories of behaviors. There is an established strong association between fertility intentions and the odds of giving birth (Schoen et al., 1999; Shreffler et al., 2016). Fertility intentions, however, do not provide consistent predictions of achieved, or completed, fertility at either the individual or aggregate level (Beaujouan & Berghammer, 2019; Quesnel-Vallée & Morgan, 2003, Shreffler, 2024). Inconsistency in predicting fertility outcomes from fertility intentions has led to questions about the utility of using mostly measures of fertility intentions to predict the odds of a birth (Bongaarts, 2001). Motivational schemas about motherhood or the value of children are conceptualized as occurring prior to fertility intentions (Miller, 2012), but while they are strongly correlated, they are distinct constructs (McQuillan et al., 2015). Therefore, it is important to explore how concepts such as importance of motherhood, considered a trait or desire, to better understand the odds of first and subsequent birth outcomes. Our goal is to advance prediction of a first or subsequent birth beyond reliance upon intentions.

To reach this goal, we analyze Waves 1 and 2 of the U.S. National Survey of Fertility Barriers (NSFB). Specifically, we focus on a subsample of women who are not surgically sterile to estimate associations among fertility intentions and importance of motherhood at Wave 1 with fertility outcomes by Wave 2 (approximately three years later), controlling for theoretically relevant variables. Our expectation is that including importance of motherhood in our analysis will account for additional variance compared to a model that does not include importance of motherhood. We suggest that including importance of motherhood in studies of fertility outcomes will allow researchers to make more accurate assessments. We suspect that the relationship between importance of motherhood and having a subsequent birth will be stronger for women without children compared to women with children.

## 2. Theoretical framework

### 2.1. Conceptualizing and measuring fertility intentions

Because fertility behavior often involves a certain degree of planning, demographers typically include measures of fertility intentions, ideals, and desires in theoretical models of human fertility (van de Walle, 1992). Recent conceptualizations about fertility intentions often place them within broader social and cognitive contexts (Bachrach & Morgan, 2013). In the Theory of Planned Behavior (TPB), Ajzen and Klobas (2013) conceptualize fertility intentions as proximate causes, whereas desires, preferences, and ideals are generally viewed as distal causes of fertility behavior (Hin et al., 2011). The Traits-Desires-Intentions-Behavior (TDIB) theoretical framework (Miller, 2012; Miller & Pasta, 1993) suggests that there is a sequential process to understanding fertility behavior. Motivational traits are informed by individual characteristics and experiences; desires refer to a more specific number of children an individual wants to have; intentions are formed when an individual is ready to act on desires; and behaviors closely follow intentions to act (Miller, 2021). In this conceptualization, the progression from traits through desires and intentions to behavior represents a path from more stable characteristics of the individual to situational characteristics shaped by life course cues and social structural realities.

The Theory of Conjunctural Action (Bachrach & Morgan, 2013; Johnson-Hanks et al., 2011; Rackin & Bachrach, 2016) builds upon prior theories of fertility (e.g., TDIB, TPB) by combining them with considerations drawn from the life-course perspective, cognitive science, and systems theory. TCA views fertility behavior as the result of both conscious intentions and emotionally laden “automatic” cognitions. This perspective views cognitions as deriving from social structures, which are conceptualized as including both material structures (e.g. policies) and schemas (e.g., values, beliefs, norms, scripts, and ways of categorizing). In this framework, schemas function as a lens through which social context, norms, and proscriptive behavior are connected to childbearing (Bachrach & Morgan, 2013; Johnson-Hanks et al., 2011).

Questions about fertility intentions are common in demographic surveys. Fertility intentions are measured retrospectively by asking women if a particular pregnancy or birth was intended or prospectively by asking women if they intend to have another child and then following up to assess whether a birth occurred (Rackin & Morgan, 2018). Social desirability can bias retrospective measures of intentions towards intended pregnancies because mothers may engage in postpartum rationalizations. Prospective measures can overestimate the unintended pregnancy rate because of the instability of fertility intentions (i.e., women changing their minds) (Rackin & Morgan, 2018). Retrospective measures might be best suited to assess unintended pregnancies (Rackin & Morgan, 2018), whereas prospective measures are more appropriate when predicting future fertility behavior (Moreau et al., 2012).

Rather than predict whether a particular birth was intended, some studies focus on the total desired number of children (Morgan & Rackin, 2010; Ni Bhrolcháin & Beaujouan, 2011). Other studies measure whether or not people intend to have any (more) children, thus creating a dichotomous outcome. Some surveys specify a time limit regarding intention or desire to have a child (e.g., “in the next three years”) (Testa et al., 2016), and other surveys leave the time horizon open-ended (Miettinen et al., 2011). In a meta-analysis of studies examining the relationship between education and fertility intentions for different parity levels, Testa (2017) found that, of 86 analyses, 73 % used open-time horizon measures of intentions, 20 % used time-limited measures, and 7 % used measures of intended parity.

Researchers sometimes assume that all women have clear intentions, but many women are uncertain or ambivalent about their intentions (Bernardi et al., 2015; Kost & Lindberg, 2015). Women who report that they are unsure about their intentions could be transitioning between intentions (Lifflander et al., 2007) or be less intentional regarding their fertility (McQuillan et al., 2011). Preference Construction Theory (NíBhrolcháin & Beaujouan, 2015) posit that women construct their fertility preferences based on their current circumstances at the time of the survey, suggesting that uncertainty and shifting preferences are common. It is now common for surveys of fertility behavior to include questions about the certainty of intentions (Bachrach & Newcomer, 1999; Santelli et al., 2009), and indicators of the certainty of intentions are strong predictors of subsequent fertility behavior (Spéder & Kapitány, 2009).

### 2.2. Fertility intentions and the life course

Recent empirical evidence indicates that women change their fertility intentions over the life course with changing social contexts (Iacovou & Tavares, 2011; Ni Bhrolcháin et al., 2010; Hayford, 2009; Kuhnt et al., 2021; Shreffler et al., 2015). The life course perspective (Elder et al., 2003) provides a useful framework for explaining variation in fertility intentions with changes in life circumstances (e.g., finishing school, gaining or losing a partner). The life course perspective emphasizes how behaviors are shaped by historical and biographical constraints and opportunities and thus suggests the importance of including contextual, individual, and social factors in models of the associations between fertility intentions and subsequent births.

Life course norms associated with social cues (i.e., marriage, education, career stability) help to shape when women in the U.S. have children (McMahon, 1995). The two-child norm in the U.S. (Hagewen & Morgan, 2005) and long-held attitudes that large and one-child families are less desirable (Thornton & Young-DeMarco, 2001) are also wide-spread norms that shape childbearing. Fertility intentions can change with each child, leading some to assert that fertility intentions are a “moving target” (Hayford, 2009; Quesnel-Vallée & Morgan, 2003). Using a nationally representative sample of childbearing-aged women, Shreffler et al. (2015) found that the majority of women (60 %) reported differing intentions across their pregnancies, suggesting that intendedness is often pregnancy-specific.

### 2.3. Motivations for motherhood

Another line of research has focused on reasons why people want children. Economic and demographic research often focuses on the costs and benefits associated with having children (Morgan & King, 2001; Schoen et al., 1997). Higher opportunity costs (e.g., time out of the labor force, childcare) contribute to lower fertility in high-income countries (Gerson, 2009; Mills et al., 2011). Despite the economic hardships associated with raising children, however, most people still desire at least two children (Morgan & Rackin, 2010). Researchers have struggled to explain the apparent inconsistency between the high cost of children and the high desire for children (Morgan & King, 2001). Edin and Kefalas (2005), who studied Black and Hispanic single mothers, suggest that the decision to become a mother is neither simply based on a financial calculus or a normative marriage but reflects other important social meanings, desires, and attitudes as well.

There are non-economic benefits to parenting, including emotional rewards, which can outweigh material costs. Caldwell (1982) argued that women have children despite the costs because “…one’s own children provide a unique form of pleasure which is not substitutable” and because “they can afford that expenditure for such a unique pleasure.” The women that Edin and Kefalas (2005) studied in urban Philadelphia said that life without children is meaningless, despite the tremendous extra economic burdens children present to those in economically strained circumstances. In addition, although the “Motherhood Mandate” has weakened over the past several decades (Koropeckyj-Cox & Pendell, 2007), pronatalist pressures remain, although their strength and influence vary by social location and culture (McQuillan et al., 2015).

Fertility studies in recent decades have incorporated the perceived value of children (e.g., costs and benefits) to enable assessment of whether these perceptions matter for fertility outcomes (Liefbroer, 2005; Thomson, 2015). The National Survey of Families and Households (NSFH) assesses “attitude toward parenthood” through agreement or disagreement with the statement: “It is better to have a child than to go through life childless” or “A man/woman can have a fulfilling life without children” (Koropeckyj-Cox & Pendell, 2007). These measures approximate the “importance of motherhood” concept we examine but are usually analyzed as single indicators, therefore suffering from reduced reliability.

The *Value of Children* (VOC) scale, first developed by Hoffman and Hoffman (1973) and later modified by Nauck (2007), is primarily focused on explaining inter-societal differences in motivations for having children rather than on differences among individuals within a society (Nauck, 2014). Nauck (2007), for example, uses a modified VOC scale to compare societies with regard to the extent to which members endorse three clusters of values of children: comfort, stimulation, and social esteem. Although the VOC has been used as a summative scale (Hoffman et al., 1978), it is more often used – as in the example above – as a means of assessing the reasons *why* women want children than as a means of assessing how important motherhood is to individual women.

A second tradition of research related to the importance of motherhood has involved the use of the Childbearing Questionnaire (CBQ) (Miller, 1995) to measure positive and negative motivations for having children. The items in the CBQ overlap somewhat with the importance of motherhood concept but it also touches on concepts beyond the scope of the importance of motherhood concept, such as the experience of pregnancy. Furthermore, the CBQ has 49 items whereas the importance of motherhood scale has only five items.

The importance of motherhood scale was developed by McQuillan et al. (2008) in response to the need for a brief unidimensional scale to measure women’s personal valuation of motherhood (e.g., “Having children is important to my feeling complete as a woman,” “I think my life will be or is more fulfilling with children,” and “It is important for me to have children”). Importance of motherhood varies considerably among women and is associated with importance of work, employment, religiosity, parity, and race/ethnicity but not age and social class (McQuillan et al., 2008). McQuillan et al. (2015) found that importance of motherhood was strongly associated with fertility intentions and served as a partial mediator between variables such as personal attitudes, social pressure, parity, socioeconomic status, and race/ethnicity, on the one hand, and fertility intentions, on the other. This suggests the need to explore whether importance of motherhood constitutes an aspect of the motivational frame that can shape fertility behavior, and, if this is the case, to determine the extent to which it is mediated by fertility intentions.

### 2.4. The relationship between motivations and intentions

Importance of motherhood would likely be classified as a “desire” by the TPB approach, a “trait” by the TDIB approach, and a “schema” by the CTA approach. However it is named, we suspect that the importance of motherhood constitutes part of the motivational frame that is both logically and temporally prior to the formation of intentions. Survey questions measuring fertility intentions capture some of the rational dimensions of having children, but they do not always capture the motivational frame concerning motherhood (Mumford et al., 2016). Preferences and attitudes are rarely included in models predicting births despite evidence that they contribute substantially to realized fertility (Barber & Axinn, 2005). Women vary in the importance they place on motherhood (McQuillan et al., 2008), but only recently has importance of motherhood been incorporated into fertility research (McQuillan et al., 2015).

We conceptualize two primary ways that importance of motherhood could influence birth outcomes. First, the effect of importance of motherhood on fertility behavior might be fully mediated by fertility intentions. As noted above, even adjusted for numerous characteristics, there is an association between importance of motherhood and fertility intentions (McQuillan et al., 2015). If the effect of importance of motherhood on fertility behavior is fully mediated by fertility intentions, then adding importance of motherhood to models of realized births **will not** add explanatory value to models predicting births. A second possibility is that importance of motherhood has a direct effect on fertility behavior over and above its influence through fertility intentions. If this is the case, adding importance of motherhood to models of realized births **will** add explanatory value to models predicting births.

We expect that an association between importance of motherhood and having a birth will be stronger for nulliparous women than it is for women with children. In fact, it is possible that there will be no association at all between importance of motherhood and having a birth, particularly if it reflects being a mother rather than motivating having children. While it seems quite likely that women who value motherhood will try to become mothers, there is no reason to expect that importance of motherhood will be associated with the number of children a woman has. The fertility intentions of younger women are more influenced by normative expectations, whereas older women are more influenced by practical considerations and contextual constraints (Hagewen & Morgan, 2005; Morgan & Rackin, 2010). Likewise, we expect that fertility intentions of nulliparous women will be more influenced by normative expectations and general preferences, while women with children will be more influenced by practical considerations and constraints. In addition, some women with higher importance of motherhood may prefer to have fewer children in order to be able to provide intensive mothering (Hays, 1996; Lareau, 2003).

## 3. Methods

### 3.1. Data and sample

This study uses the two-wave (2004–2006 and 2008–2010) U.S. National Survey of Fertility Barriers (NSFB), a random digit dialing telephone survey of 4712 women of childbearing aged (25–45) in Wave 1 and a subset of their husbands/partners. The study was designed to assess social and health factors associated with reproductive choices and fertility. The data are nationally representative after weighting to adjust for oversamples of Black and Hispanic women plus women with fertility problems. Detailed methodological information is available at https://www.icpsr.umich.edu/icpsrweb/DSDR/studies/36902#bibcite.

An attempt was made to re-interview a subsample of main respondents and all partners three years after their original interview. Wave 2 yielded 2136 main respondent interviews, roughly 58 % of those sought. To check whether attrition between waves was likely to have biased results, we conducted a logistic regression analysis of the association of fertility intentions, importance of motherhood, and racial/ethnic group with the odds of completing the second wave survey. We found that all groups (Black, Hispanic, and “Other”) had lower odds of completing Wave 2 than those who identified as white. Fertility intentions and importance of motherhood were not associated with completing Wave 2. We therefore do not think that selective attrition substantively biased our results. The analytic sample for this study is restricted to 1381 women who participated in both waves of data and were not surgically sterilized or pregnant at the time of their first interview.

### 3.2. Measures

#### Dependent variable.

The focus of this analysis is on *birth outcome*, which is measured at Wave 2 with an indicator variable (1 = birth and 0 = no birth between waves). It is possible that some women could have had pregnancies between waves that ended in miscarriage, abortion, or still birth. Such cases would be placed in the “no birth” category.

#### Independent variables.

The NSFB uses only an open time-horizon measure of fertility intentions and degree of certainty of completing fertility. The measure for *fertility intentions* is based on two questions measured at Wave 1 that are combined to create an ordinal measure of strength of fertility intentions. The first question was “Do you intend to have a baby?” and the second question was “Of course, sometimes things do not work out exactly as we intend them to, or something makes us change our minds. In your case, how sure are you that you will/will not have a child?” Possible answers to the second question were “very sure,” “pretty sure,” and “not very sure.” Responses to the combined intentions question were coded so that they range from low scores, “Very sure do not intend” (−3) to high, “Very sure do intend” (+3). Women who said they “don’t know” their intentions, who said they cannot have children, or who said they would let God or nature decide are coded 0 (the center of the scale). Women who said that they cannot have children were left in the model because some of these women may, in fact, have children (Shreffler et al., 2016). It should be noted that the women we placed in the center of the scale may be qualitatively different from women who actually reported some level of intentions insofar as the idea of planning pregnancies may not be salient to them.

The *importance of motherhood* scale was developed by McQuillan et al. (2008) and is calculated using the mean of responses to five questions, with a range of 1–4 where 4 indicates high importance of motherhood. Four items have Likert-type scales (strongly agree to strongly disagree): (1) “Having children is important to my feeling complete as a woman,” (2) “I always thought I would be a parent,” (3) “I think my life will be or is more fulfilling with children,” and (4) “It is important for me to have children.” The response categories for the fifth question (5) “How important is each of the following in your life… raising children?” range from not very important to very important. The Cronbach’s alpha coefficient (α =.77) indicates high internal consistency. We use the Wave 1 measure of importance of motherhood.

We analyzed the data separately for women without (parity=0) and with (parity 1 +) children at Wave 1. We expect that importance of motherhood will have a stronger association with a first birth than subsequent births. In addition, it is likely that at least some of the control variables’ associations will differ by parity.

#### Control variables.

We included *race/ethnicity*, *age, education*, and *religiosity* in the analysis because they are known to influence or vary in terms of fertility intentions, importance of motherhood, and realized births. We assessed *race/ethnicity* by creating three indicator variables: Black, Hispanic and other compared to non-Hispanic White, the reference category (hereafter referred to as “White”). Because participants could select more than one racial/ethnic category, we used a decision rule to categorize participants. If participants selected Hispanic and another category, they were categorized as Hispanic. We did not have sufficient sample size to make meaningful subcategories from the “other” category; we therefore simply control for this category in the models. These indicator variables were measured at Wave 1.

Age was measured in years and centered for the multivariate analyses. To allow for the possibility of a non-linear association, we also include a measure of age-squared. Education was coded into several indicator variables for high school diploma or less, some college, and a bachelor’s degree or higher. Religiosity was measured at Wave 1 by averaging the standardized versions of three questions: 1) “About how often do you pray,” 2) “How close do you feel to God most of the time,” and 3) “In general, how much would you say your religious beliefs influence your daily life?” *Region of residence* was included as a control variable because birth rates vary by state. Respondents’ zip codes were coded into four regions of the country: Northeast, West, South, and Midwest. These variables were measured at Wave 1.

Due to people’s sensitivity to questions about income, *family income* was asked based upon categories in an ordinal scale ranging from 1 (less than $5000) to 12 ($100,000 or more). We then substituted the midpoint of each category in dollar amount to convert this into a continuous scale. We use the measure of income that was asked at Wave 1. We considered using a measure of change in total family income between Wave1 and Wave 2 as our income variable, but we elected not to do this because it was not possible with this data set to determine whether a change in income came before or after a birth*.*

*Economic hardship, work hours*, and *union status* were also included as control variables because of their known associations with fertility intentions, importance of motherhood, and realized births. Responses to three questions in each wave are combined into a scale to measure economic hardship: (1) “During the last 12 months, how often did it happen that you had trouble paying bills?,” (2) “During the last 12 months, how often did it happen that you did not have enough money to buy food, clothes, or other things your household needed?,” and (3) “During the last 12 months, how often did it happen that you did not have enough money to pay for medical care?” This is a unidimensional scale with high reliability (α =.82 in Wave 1). Higher values indicate greater hardship. We calculated change in economic hardship by subtracting the economic hardship score at Wave 2 from the Wave 1 economic hardship score.

*Work hours* is based on the question, “I’d like to know a little bit about your present job. Last week, were you employed full time, going to school, keeping house, or something else. Full time employment was coded as “1.” Part time employment was coded as “2,” and all other non-missing answers were considered to indicate no paid work and were coded as “3.” This variable was measured at both waves. We calculated change in work hours by comparing answers from W1 and W2. The resulting variable was score as “1” if there was no change between waves, “2” if work hours decreased between waves, and “3” if work hours increased between waves. This variable was treated as a categorical variable with “no change” as the reference category.

Relationship transitions were measured as continuously partnered, continuously single, changing from single to partnered, or changing from partnered to single. All continuous variables were mean-centered (i.e., we subtracted the mean from all values so that “0” indicates the average value) as a way to simplify the interpretation of the constant.

### 3.3. Plan of analysis

Five variables used in our analysis had missing data: education at Wave 1 (1 case), total family income (80 cases), economic hardship change (3 cases), employment change (1 case), and union status change (4 cases). In order to avoid losing cases, we used the multiple imputation suit of commands in Stata 19 to handle missing data.

Our analysis proceeded in several steps. We first estimated the correlation between fertility intentions and importance of motherhood to discover how closely these two focal independent variables were related. We then compared the two parity groups using descriptive statistics (percentages for categorical variables and means and standard errors for continuous variables) for all of the variables in the analyses. We tested for significant differences in births, fertility intentions, importance of motherhood, and all other variables by parity at Wave 1. We used OLS regression for continuous variables and logistic regression for categorical variables.

Because birth or not between waves is a binary outcome, we use logistic regression for the multivariate analyses. As noted above, we conducted separate analysis by parity. Model 1 in each analysis provides estimates of the associations of all variables except the two focal independent variables with a birth between waves, adjusted for control variables. Model 2 includes the measure of fertility intentions to see whether it adds information to the model predicting a birth between waves (i.e., realized fertility). Model 3 replaces the measure of fertility intentions with the measure of importance of motherhood to assess the strength of the association not accounting for fertility intentions. In the final model, Model 4, we include both of the focal independent variables to assess if they have unique associations with a subsequent birth or if multicollinearity eliminates the associations.

## 4. Results

A necessary first step in exploring the relationship between importance of motherhood and fertility intentions is to estimate the correlation coefficient of these two variables. For the sample as a whole, the correlation is.246, suggesting that these variables represent related but distinct concepts. The correlation is much stronger among zero parity women (.663) and much weaker among women with children (.085).

Table 1 presents descriptive statistics for the sample as a whole and for parity groups separately. The final column indicates significant differences between women without children and women with at least one child for all variables in the analysis; we summarize differences that reach statistical significance at the.05 level unless otherwise noted. Overall, 22 % of the women (N = 1361) gave birth during the three years following the initial interview. Mean fertility intentions at Wave 1 were just below the neutral category, between “do not intend”, “not very sure” and “unsure/do not know” (−.21). There were no significant differences by parity in the percentage of women giving birth between waves. Among women with zero parity, fertility intentions were higher and importance of motherhood was lower than the averages for women with children. On average, women in the sample reported relatively high importance of motherhood (M=3.8 out of 5). More women with than without children identified as Hispanic.

Many of the women in this sample were highly educated (46 % reported at least a bachelor’s degree). Women without children were more likely to have a college degree or higher than women without children. Women with children reported higher mean religiosity scores than women without children. Most women (71 %) did not change their work hours between waves. A higher percentage of women with children increased their work hours over time compared to women without children. Women without children were more likely to be continuously single and to transition from single to partnered than women with children, but they were less likely to be continuously partnered. There were significant differences in the proportion of women who were continuously single across both waves by race/ethnicity. Because so many variables differ significantly by parity and because we have theoretical reasons to expect the effect of importance of motherhood on the probability of a birth between waves to vary by parity, we conduct separate multivariable analyses for each group.

Table 2 displays the results of the logistic regression models of births between waves by parity. The first set of models is restricted to women who have parity zero. Model 1 displays predicted probabilities of a birth between waves for all variables except the focal independent variables. The McFadden’s R-squared value of 27.9 indicates an improved model fit predicting the probability of a birth between waves. Among women with zero parity, being older, being more religious, having higher income, and decreasing work hours were associated with increased probability, while being continuously single was associated with lower probability of having a birth between waves. We add fertility intentions to Model 2, which improves the model fit to 34.5. Among women without children, each unit increase in intentions results in a.06 increase in the probability of having a birth between waves.

In Model 3 we add importance of motherhood to the model and remove fertility intentions. This model has a better fit compared to Model 2 (36.2 compared to 34.5). Among women without children, each unit increase in importance of motherhood results in a.16 increase in the probability of having a birth between waves. Note that the effect of the importance of motherhood on the probability of a birth between waves in Model 3 is greater than the effect of fertility intentions on the probability of a birth between waves in Model 2.

Model 4 includes both fertility intentions and importance of motherhood in the same model. This model provides the best fit for explaining the variation in the probability of a birth between waves (37.8). Among women without children, each unit increase in importance of motherhood results in a.16 increase in the probability of having a birth between waves. The effect of a one unit increase in the importance of motherhood on the probability of a birth between waves (.11) is greater than the effect of a one unit increase in fertility intentions on the probability of a birth between waves (.03). This difference is significant at the.05 level (t = 2.42).

The second set of models is restricted to women with children. Model 1 displays predicted probabilities of a birth between waves for all variables except the focal independent variables. This model reflects a better fit than the intercept model with a McFadden’s R-squared value of 23.1, lower than the model fit value for zero parity women (27.9). Model 2 adds fertility intentions to the model. Adding fertility intentions to the model increases model fit to 32.6. Among women with children, each unit increase in intentions results in a.05 increase in the probability of having a birth between waves.

Model 3 adds importance of motherhood to the model and removes fertility intentions. The fit for Model 3 compared to Model 2 is lower (23.4 compared to 32.6). Among women with children, an increase in importance of motherhood has no significant effect in the probability of having a birth between waves. Among women with children, the effect of the importance of motherhood on the probability of a birth between waves in Model 3 is considerably less than the effect of fertility intentions on the probability of a birth between waves in Model 2.

Model 4 includes both fertility intentions and importance of motherhood in the same model. This model provides a better fit estimating the probability of a birth between waves (37.8) than estimating with fertility intentions or importance of motherhood alone. The model fit is comparable with Model 2 (32.8 compared to 32.6). Among women with children, each unit increase in fertility intentions results in a.05 increase in the probability of having a birth between waves. The effect of a one unit increase in fertility intentions on the probability of a birth between waves is not significantly greater than the effect of a one unit increase in the importance of motherhood on the probability of a birth between waves. (t = −.07).

## 5. Discussion and conclusion

Consistent with fertility theories – TPB, TDIB, and CAT – that emphasize the importance of schemas and motivations rather than simply intentions (Brehm & Schneider, 2019; Johnson-Hanks et al., 2011), we assessed the utility of adding importance of motherhood to models of subsequent births that already include fertility intentions. We make two valuable contributions to research on fertility intentions and subsequent births. First, even though recent qualitative studies and fertility theories emphasize the importance of schemas and motives for understanding birth outcomes, our study is the first to directly include an importance of motherhood scale in a study predicting subsequent births among women. McQuillan and colleagues (2015) found that higher importance of motherhood scores are associated with higher fertility intentions, giving rise to the possibility that both measures might be capturing the same underlying construct. As hypothesized, however, we found that each measure provides unique information in models of first births. Thus, we provide support for including the importance of motherhood in efforts to understand subsequent births.

Second, as hypothesized we found that the associations between fertility intentions, importance of motherhood, and fertility outcomes differ by parity. Importance of motherhood accounts for a good deal of the variation in subsequent births among women without children, but it accounts for very little of the variation in subsequent births among women with children. In fact, our findings suggest that importance of motherhood may be an even stronger predictor of having a birth than fertility intentions among women without children. This finding aligns with our expectations. It seems obvious that women who value motherhood would be more likely to have at least one child as compared to those who do not highly value motherhood, but it is unclear if importance of motherhood should lead to having more children or if it means providing more intensive mothering to the same number or fewer children.

### 5.1. Limitations and future directions

The study has several limitations for data and measurement reasons that can guide directions for future research. First, the measure of fertility intentions included women who claimed not to have fertility intentions. We noted above that some women may simply not endorse the notion that pregnancy should be planned (Greil et al., 2023) and should therefore be treated as qualitatively different from women who are willing to state intentions. Therefore, future research might benefit from the inclusion of attitudes about the importance of planning pregnancies in studies of the association between fertility intentions and birth outcomes. Also, the measure of fertility intentions included in the NSFB captures an open-ended time horizon for intentions as opposed to short-term intentions, which are more accurate for predicting births (Morgan, 2001). The short window between NSFB study waves of only three years also is a limitation as births might have happened after the second wave was completed. Using different measures of intentions with more waves of data collection over a longer time window might well result in different findings than those reported here.

Intersectional theorizing about motherhood (e.g., Collins, 1990; Taylor, 2011) suggests that future research with a larger sample should explore whether combinations of indicators of social location (e.g., race/ethnicity, socioeconomic status, sexual minority status, and immigration status) shape the association of fertility intentions or importance of motherhood and subsequent births. The NSFB is large and nationally representative and has measures of both fertility intentions and attitudes toward the importance of motherhood, but the dataset does not have sufficient cases for more refined analysis of intersecting groups.

Additionally, the NSFB did not include a measure of contraception access, which limits our ability to consider the mediating role of contraception for the relationship between intentions, attitudes, and subsequent births. In this study, we did not differentiate between intended, unintended, wanted, nor unwanted births. Disparities in access and effective utilization of contraceptives may explain some of the disparities in unintended pregnancy by race/ethnicity and socioeconomic status (Hall et al., 2012). A study of women in St. Louis found that reducing costs and obstacles preventing LARC uptake eliminated racial disparities in unintended pregnancy (Goodman et al., 2017). A public health focus on reducing unintended pregnancies continues to emphasize contraception as a primary tool for reducing disparities, but contraceptive use and nonuse is likely also influenced by attitudes toward pregnancy planning, intentions, and emotional reactions to a potential pregnancy (Gomez et al., 2018; Grady et al., 2015; Hayford & Guzzo, 2013).

Despite the study limitations, we believe the findings reveal important insights about the role of attitudes about motherhood for fertility outcomes among individuals. There has been considerable attention on the utility of fertility intentions measures for the prediction of fertility outcomes for decades (Shreffler, 2024). Our findings suggest that the meaning of motherhood may actually be a more important factor for predicting births than fertility intentions among women without children. Future studies of fertility and fertility decision-making would be well advised to include importance of motherhood and/or other measures of traits, desires, and motivations beyond intentions.

## Figures and Tables

**Table 1:** Weighted descriptive statistics by parity (N = 1381).

	Total		Parity 0		Parity 1 +		
	M/%	SE	M/%	SE	M/%	SE	SIG
Birth Between Waves	.22	.01	.22	.02	.21	.02	
Fertility Intentions at W1	−.21	.08	.35	.11	−.52	.10	***
Importance of Motherhood W1	3.21	.02	2.80	.04	3.44	.02	***
Race/Ethnicity Categories							
Non-Hispanic White	.68	.02	.71	.02	.67	.02	
Non-Hispanic Black	.12	.01	.12	.01	.11	.01	
Hispanic	.12	.01	.08	.01	.15	.02	***
Non-Hispanic Other	.08	.01	.10	.02	.07	.02	
Age	33.98	.20	32.47	.29	34.79	.25	
Education at Wave 1							
HS Diploma or Less	.27	.02	.17	.02	.33	.02	***
Some College	.27	.01	.20	.02	.31	.02	***
BA Degree or Higher	.46	.02	.64	.03	.36	.02	***
Region							
Northeast	.15	.01	.18	.02	.13	.02	
Midwest	.26	.01	.27	.02	.25	.02	
South	.34	.02	.32	.02	.35	.02	
West	.25	.01	.23	.02	.26	.02	
Religiosity W1	−.15	.03	−.48	.05	.02	.04	***
Total Family Income W1 (in thousands)	43.14	.98	44.42	1.50	42.44	1.27	
Economic Hardship Change	.03	.03	−.04	.04	.06	.03	*
Work Hours							
Work Hours Decrease	.15	.01	.17	.02	.13	.02	
Work Hours Same	.71	.02	.72	.02	.71	.02	*
Work Hours Increase	.14	.01	.11	.02	.16	.02	*
Union Status Change							
Continuously Single	.20	.01	.33	.02	.14	.02	*
Continuously Partnered	.67	.02	.49	.03	.77	.02	***
Partnered to Single	.06	.01	.05	.01	.06	.01	
Single to Partnered	.07	.01	.13	.02	.04	.01	***
N	1381		538		843		

Data: Wave 1 and Wave 2 (2004–2006) National Survey of Fertility Barriers.

Note: Means for continuous variables; Percentages for categorical variables.

Note

* > .05;

** > .01;

*** > .001

**Table 2:** Predicted probabilities from logistic regression models estimating birth between waves for zero (N = 538) and 1 + Parity (N = 843).

	Zero Parity Only												Parity 1 + only											
	Model 1			Model 2			Model 3			Model 4			Model 1			Model 2			Model 3			Model 4		
	dy/dx		SE	dy/dx		SE	dy/dx		SE	dy/dx		SE	dy/dx		SE	dy/dx		SE	dy/dx		SE	dy/dx		SE
Fertility Intentions W1 (centered)				.06	***	.01				.03	**	.01				.05	***	.01				.05	***	.01
Importance of Motherhood W1 (centered)							.16	***	.02	.11	***	.03							.05		.03	.05		.03
Race/Ethnity																								
Black	.03		.06	.00		.06	.05		.06	.04		.06	−.01		.05	−.02		.05	.01		.05	.00		.05
Hispanic	.08		.07	.05		.05	.11		.06	.08		.05	.08		.04	.07		.05	.09	*	.05	.08		.05
Race-Other	.23	**	.08	.17	*	.07	.15	*	.07	.15	*	.07	−.11	*	.05	−.13	**	.04	−.11	*	.05	−.12	**	.04
Age(centered)	.19	***	.05	.16	***	.04	−.60		.71	−.62		.72	.02		.04	.04		.04	.01		.04	.04		.04
Age (centered)Sq	.00	***	.00	.00	***	.00	.01		.01	.01		.01	.00		.00	.00		.00	.00		.00	.00		.00
Education at Wave 1																								
Some College	−.10		.07	−.14	*	.06	−.11		.06	−.12	*	.06	−.03		.04	−.06		.04	−.03		.04	−.06		.04
BA Degree or +	−.01		.06	−.08		.05	−.01		.05	−.05		.05	.08	*	.03	.01		.04	.07	*	.04	.00		.04
Region																								
Midwest	−.02		.06	−.01		.05	−.02		.05	−.01		.05	.01		.05	−.03		.04	.01		.05	−.02		.04
South	−.06		.06	−.05		.05	−.07		.05	−.06		.05	.04		.05	.02		.05	.04		.05	.02		.05
West	−.05		.06	−.06		.05	−.05		.06	−.05		.05	.01		.05	−.02		.05	.02		.05	−.01		.05
Religiosity W1	.04	*	.02	.03		.02	.02		.02	.02		.02	.01		.02	.02		.02	.01		.02	.01		.02
Family Income W1 (centered)	.00	*	.00	.00		.00	.00	*	.00	.00	*	.00	.00		.00	.00		.00	.00		.00	.00		.00
Economic Hardship Change (centered)	.04		.03	.05		.03	.04		.03	.04		.03	.04		.02	.03		.03	.03		.02	.03		.02
Employment Change																								
Resp Work Hours- Decrease	.13	**	.04	.09	**	.03	.10	*	.04	.08	*	.03	.04		.04	.04		−.04	−.03		.04	−.03		.04
Resp Work Hours- Increase	−.07		.05	−.06		.05	−.06		.05	−.05	**	.04	−.10	**	.03	−.08	*	.03	−.10	**	.03	−.08	*	.03
Union Status Change																								
Continuously Single	−.28	***	.03	−.26	***	.03	−.27	***	.03	−.26	***	.03	−.19	***	.03	−.18	***	.04	−.18	***	.03	−.17	***	.04
Partnered to Single	.00		.10	−.03		.08	.01		.08	−.02		.08	−.19	***	.03	−.18	***	.04	−.19	***	.04	−.18	***	.04
Single to Partnered	−.04		.06	−.02		.06	−.05		.06	−.04		.05	.05		.10	−.01		.10	.05		.10	−.02		.10
*N*	538			538			538			538			843			843			843			843		

Data: Wave 1 and Wave 2 (2004–2006) National Survey of Fertility Barriers.

Note

* > .05;

** > .01;

*** > .001
